# *Aedes aegypti* mosquitoes from Guadeloupe (French West Indies) are able to transmit yellow fever virus

**DOI:** 10.1371/journal.pone.0204710

**Published:** 2018-09-28

**Authors:** Pei-Shi Yen, Fadila Amraoui, Anubis Vega Rúa, Anna-Bella Failloux

**Affiliations:** 1 Institut Pasteur, Department of Virology, Unit of Arboviruses and Insect Vectors, Paris, France; 2 Institut Pasteur de Guadeloupe, Environment and Health Unit, Laboratory of Vector Control Research, Les Abymes, France; Centro de Pesquisas René Rachou, BRAZIL

## Abstract

The recent yellow fever epidemic in Brazil has raised the concern of outbreaks in neighboring countries, particularly in the Caribbean region where the vector *Aedes aegypti* is predominant. This threat comes from the past when in the Americas, this disease caused devastating urban epidemics. We report the vector competence of *Ae*. *aegypti* from Guadeloupe for yellow fever virus by determining different parameters describing virus infection, dissemination, and transmission. The results indicate that *Ae*. *aegypti* Guadeloupe are susceptible to yellow fever virus with viral particles detected in mosquito saliva at 14 and 21 days post-infection. Local authorities and more broadly, international organizations should maintain the active surveillance of *Aedes* mosquitoes and the spreading of human cases from South America.

## Introduction

Yellow fever (YF) is a mosquito-borne viral disease endemic to some countries of South America and sub-Saharan Africa. It can present various clinical features ranging from a self-limited, mild febrile illness to fatal symptoms such as hemorrhages and liver damages. Most of all cases reported annually (80–90%) occur in Africa where YF covers 44 countries [1]. In South America, YF is described in less than 10 countries: Argentina, Bolivia, Brazil, Colombia, Ecuador, Paraguay, Peru, and Venezuela (http://ais.paho.org/phip/viz/ed_yellowfever.asp). In these locations, YF uses to periodically spread via epizootic outbreaks following the displacements of non-human primates [2]. From July 2017 through March 2018, the states of Rio de Janeiro, Minas Gerais, and São Paulo in Brazil, counted 932 human cases including 300 fatal cases [3]. Alarmingly, human cases were reported near São Paulo city, threatening the initiation of an urban transmission that has not been notified in the country since 1942.

Originally from Africa, YF is believed to be introduced into America via the slave trade in the middle of 18^th^ century [4]. Approximately 10.7 million slaves were deported to the Caribbean, North and South Americas during four centuries [5]. Likewise, the mosquito *Ae*. *aegypti* by finding suitable breeding sites in slave transport ships, was introduced in America at the same period [6]. Deadly YF epidemics devastated the continent for centuries. An eradication campaign targeting *Ae*. *aegypti* organized by the Pan American Health Organisation (PAHO) was initiated in 1946 and led to the elimination of the vector from most American countries, and consequently, the disappearance of urban YF [7]. Unfortunately, the eradication campaign was interrupted and most countries were re-infested by the vector [8].

Yellow fever virus (YFV; *Flavivirus*, Flaviviridae) is primarily transmitted by the mosquitoes *Aedes* spp. (e.g. *Aedes africanus)* in Africa and *Haemagogus* (e.g. *Haemagogus janthinomys)* in South America [9]. In Brazil, the anthropophilic mosquitoes *Ae*. *aegypti* and *Aedes albopictus* as well as the YFV-enzootic mosquitoes *Haemagogus leucocelaenus* and *Sabethes albiprivus* are highly susceptible to YFV [10]. Thus, the widely distributed *Ae*. *aegypti* in American countries raises the concern of a re-urbanization of YF if the virus is introduced via viremic vertebrate hosts. In the Caribbean, Guadeloupe Island has experienced several outbreaks caused by arboviruses such as dengue [11], chikungunya [12] and zika [13], all three viruses only be transmitted by *Ae*. *aegypti* as *Ae*. *albopictus* is absent from the island [14]. To be considered as an epidemic vector of YFV, *Ae*. *aegypti* should be experimentally susceptible to the virus (i.e. a competent vector) in addition of being an anthropophilic mosquito [15] in close contacts with humans [16]. In this report, we evaluate the vector competence of *Ae*. *aegypti* from Guadeloupe to YFV. These results will help the local health authorities and the decision-makers to anticipate the arrival of YF in the Caribbean.

## Materials and methods

### Virus strain

The strain IEC-4408 (YFV-4408; accession number: KY861728) belonging to the 1E lineage of YFV, was isolated from a Howler monkey in 2008 [10]. The strain was passaged four times on *Ae*. *albopictus* C6/36 cells. Viral stocks for mosquito infections were produced on C6/36 cells and stored at -80°C.

### Artificial blood feeding

Six boxes of 60 7-day-old F1 female adults (F0 collected as larvae in June 2017 in Deshaies, Basse Terre, Guadeloupe) were fed on an infectious blood meal containing 1.4 mL of washed rabbit red blood cells and 0.7 mL of virus suspension. The blood meal supplemented with ATP as a phagostimulant at a final concentration of 1 mM was provided to mosquitoes at a titer of 10^6.5^ focus-forming unit (ffu)/mL using a Hemotek membrane feeding system. Engorged mosquitoes were transferred into boxes and maintained with 10% sucrose at 28°C under a photoperiod of 12:12.

### Mosquito sampling and processing

Mosquitoes were examined at 7, 14, and 21 days post-infection (dpi). After removing mosquito wings and legs, the proboscis was inserted into a P20 tip filled with 5 μL of fetal bovine serum (FBS) [17]. After 30 min, saliva was expelled from the tip to 45 μL of L-15 medium (Invitrogen, California, USA) and then processed for viral titration to estimate transmission. Then, mosquito head and body were collected and ground individually in 300 μL of L-15 medium supplemented with 2% FBS, for respectively, viral infection and dissemination analysis. 200 μL of homogenates were collected after centrifugation at 10,000 g for 5 min at +4°C before viral titration. To estimate the vector competence, three parameters were calculated: (i) infection rate (IR) referring to the proportion of mosquitoes with infected body among engorged mosquitoes, (ii) dissemination rate (DR) corresponding to the proportion of mosquitoes with infected head among mosquitoes with infected body, and (iii) transmission rate (TR) representing the proportion of mosquitoes with infectious saliva among mosquitoes with infected head.

### Virus titration

Mosquito samples were titrated by focus fluorescent assay on *Ae*. *albopictus* C6/36 cells in 96-well plates [18]. After 5 days of incubation at 28°C, plates were stained using antibodies specific to YFV as the primary antibody and conjugated Alexa Fluor 488 goat anti-mouse IgG as the second antibody (Life Technologies, California, USA).

### Statistical analysis

Statistical analyses were performed with Stata software (StataCorp LP, Texas, and USA). P-values<0.05 were considered significant.

## Results

**Fig 1:**
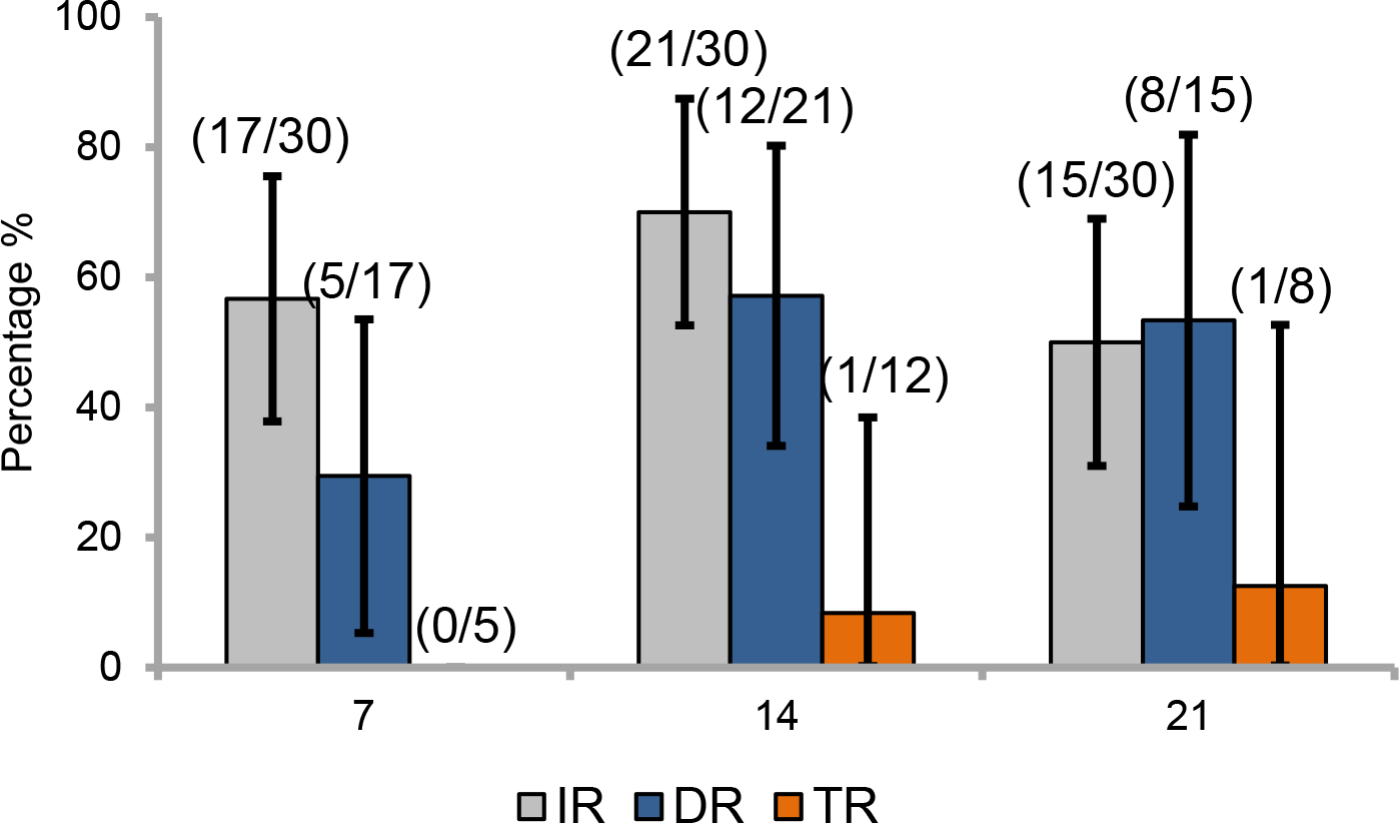
Infection, dissemination and transmission rates of *Aedes aegypti* guadeloupe to YFV (IEC-4408, 1E lineage). Mosquitoes were exposed to an infectious blood meal with YFV provided at a titer of 10^6.5^ ffu/mL. After infection, mosquitoes were examined at 7, 14, and 21 days post-infection. Error bars show the 95% confidence interval. In brackets, the number of mosquitoes examined. IR: the proportion of mosquitoes with infected body among engorged mosquitoes; DR: the proportion of mosquitoes with infected head among mosquitoes with infected body; TR: the proportion of mosquitoes with infectious saliva among mosquitoes with infected head.

Mosquitoes were analyzed at three time points following infection: 7, 14 and 21 days post infection (dpi). At 7 dpi, 56.7% (17/30) of mosquitoes examined had infected bodies (Fig 1). Among them, 29.4% (5/17) of mosquitoes were able to ensure a viral dissemination beyond the midgut barrier in the hemocele. No mosquitoes were able to transmit the virus with no viral particles detected in mosquito saliva. Later, at 14 dpi, a higher proportion, 70% (21/30) of mosquitoes had an infected body and among them, 57.1% (12/21) presented a positive viral dissemination into the hemocele. Then, 8.3% (1/12) had virus in saliva, indicative of viral transmission; one mosquito had 20 viral particles. At 21 dpi, the proportion of mosquitoes with infected body decreased to 50% (15/30) and among them, more than half (53.3%; 8/15) could ensure a viral dissemination. A slightly higher proportion of mosquitoes (12.5%; 1/8) with disseminated infection presented virus in saliva; one mosquito had 200 viral particles. Rates did not significantly vary according to dpi: 7 dpi (Chi-square test: χ2 = 2.57, df = 2, p = 0.27), 14 dpi (Chi-square test: χ2 = 3.21, df = 2, p = 0.20), and 21 dpi (Chi-square test: χ2 = 0.65, df = 2, p = 0.72).

## Discussion

Except Trinidad and Tobago in 1979 [19], the Caribbean has not suffered from YF since 1960. The ongoing YFV circulation in Brazil raises concern regarding viral importation into the Caribbean. However, the requirement of YF vaccination is not mandatory in many Caribbean islands (e.g. Haiti, Cuba), but restricted to the travelers coming from YF-epidemic countries (http://www.who.int/ith/ITH_country_list.pdf?ua=1). The disease control relies only on the check of vaccination card, which is insufficient for YF prevention. Thus, YF is still a threat for this region where *Ae*. *aegypti* is widely distributed. Here, although not based on vertebrate animal transmission model, we demonstrated that *Ae*. *aegypti* from Guadeloupe are susceptible to YFV and able to transmit viral particles from 14 days post-infection.

Viral infection, dissemination and transmission increased along with dpi. Infection and dissemination reached a peak at 14 dpi (70% and 57% for IR and DR, respectively), suggesting that the midgut has a limited role as barrier to the viral dissemination in the mosquito general cavity. Transmission was only detected from 14 dpi suggesting an extrinsic incubation period (i.e. period between the ingestion of infectious blood meal and the excretion of virus in saliva) is between 7 and 14 days as shown for *Ae*. *aegypti* populations from Congo and Brazil [10]. However transmission was quite low (i.e. 8%) suggesting a significant role of salivary glands to retain viral particles. At 21 days post-infection, transmission was more efficient with 12% of mosquitoes presenting disseminated infection and delivering virus in saliva.

It is now widely admitted that vector competence depends on the virus genotype, the mosquito genotype and their interactions, promoting local adaptation of viral lineages to mosquito vector populations [20]. The table below (Table 1) presents the vector competence of several *Ae*. *aegypti* populations from Africa, America, Asia and South Pacific region to several lineages/genotypes of YFV, exemplifying the specific outcome to each combination virus-vector.

**Table 1:** Vector competence of different *Aedes aegypti* populations to YFV.

Mosquitoes		F^a^	Titer of blood meal	Virus strain	Day post-infection	IR^b^	DR^c^	TR^d^	Reference
Country	Locality								
Kenya	Nairobi	F1	6.7–7.5 Log_10_ pfu/mL	East African	14	7 (5/75)	0 (0/5)	ND	[21]
	Mariakani	F1	6.7–7.5 Log_10_ pfu /mL	East African	14	41 (31/75)	45 (14/31)	ND	
	Kerio Valley	F1	6.7–7.5 Log_10_ pfu /mL	East African	14	11 (8/75)	38 (3/8)	ND	
	Kakamega	F1	6.7–7.5 Log_10_ pfu /mL	East African	14	25 (19/75)	42 (8/19)	ND	
South Africa	Durban	F3	>9.5 Log_10_ MID_50_/mL	BA-55 (Nigeria, 1987)	20	ND	15 (7/48)	ND	[22]
		F2	7.9 Log_10_ MID_50_/mL	BC7914 (Kenya)	18–25	ND	2 (1/45)	ND	
	Skukusa	F2/3	>9.5 Log_10_ MID_50_/mL	BA-55 (Nigeria, 1987)	20	ND	10 (4/38)	0 (0/4)^f^	
		F1	8 Log_10_ MID_50_/mL	BC7914 (Kenya)	15–20	ND	6 (2/32)	ND	
	Pafuri	F1	7.5 Log_10_ MID_50_/mL	BA-55 (Nigeria, 1987)	20	ND	0 (0/40)	ND	
		F1	8 Log_10_ MID_50_/mL	BC7914 (Kenya)	15–25	ND	9 (4/46)	ND	
Guinea	Boulbinet	F1	10^8.7^ MID_50_/mL	74018 (Brazil, 2001)	14	ND	3 (4/123)	ND	[24]
Capo Verde	Praia	F1/2	10^7^ ffu/mL	S-79 (Senegal, 1979)	14	ND	15 (6/41)	50 (3/6)	[23]
Brazil	Rio	F1	10^6^ pfu/mL	74018 (Brazil, 2001)	21	45 (9/20)	22 (2/9)	50 (1/2)	[10]
		F1	10^6^ pfu/mL	4408 (Brazil, 2008)	21	85 (17/20)	59 (10/17)	60 (6/10)	
		F1	10^6^ pfu/mL	S-79 (Senegal, 1979)	21	60 (12/20)	75 (9/12)	22 (2/9)	
	Goiania	F1	10^6^ pfu/mL	74018 (Brazil, 2001)	21	65 (13/20)	92 (12/13)	0 (0/12)	
		F1	10^6^ pfu/mL	4408 (Brazil, 2008)	21	10 (2/20)	100 (2/2)	50 (1/2)	
		F1	10^6^ pfu/mL	S-79 (Senegal, 1979)	21	0 (0/20)	ND	ND	
	Manaus	F1	10^6^ pfu/mL	74018 (Brazil, 2001)	21	55 (11/20)	54 (6/11)	33 (2/6)	
		F1	10^6^ pfu/mL	4408 (Brazil, 2008)	21	70 (14/20)	85 (12/14)	17 (2/12)	
		F1	10^6^ pfu/mL	S-79 (Senegal, 1979)	21	35 (7/20)	14 (1/7)	100 (1/1)	
	Milhas	F1	10^8.7^ MID_50_/mL	74018 (Brazil, 2001)	14	ND	0 (0/148)	ND	[24]
	Commendador Soares	F1	10^8.7^ MID_50_/mL	74018 (Brazil, 2001)	14	ND	1 (1/110)	ND	
	Quixeramobim	F1	10^8.7^ MID_50_/mL	74018 (Brazil, 2001)	14	ND	2 (2/120)	ND	
	Rocinha	F1	10^8.7^ MID_50_/mL	74018 (Brazil, 2001)	14	ND	3 (4/121)	ND	
	Tingua	F1	10^8.7^ MID_50_/mL	74018 (Brazil, 2001)	14	ND	5 (5/103)	ND	
	Pacuja	F1	10^8.7^ MID_50_/mL	74018 (Brazil, 2001)	14	ND	6 (4/71)	ND	
	Salvador	F1	10^8.7^ MID_50_/mL	74018 (Brazil, 2001)	14	ND	6 (7/111)	ND	
	Higienopolis	F1	10^8.7^ MID_50_/mL	74018 (Brazil, 2001)	14	ND	7 (8/120)	ND	
	Moqueta	F1	10^8.7^ MID_50_/mL	74018 (Brazil, 2001)	14	ND	8 (9/118)	ND	
	Feira de Santana	F1	10^8.7^ MID_50_/mL	74018 (Brazil, 2001)	14	ND	11 (5/47)	ND	
	Rio Branco	F1	10^8.7^ MID_50_/mL	74018 (Brazil, 2001)	14	ND	11 (13/117)	ND	
	Leandro Ferreira	F1	10^8.7^ MID_50_/mL	74018 (Brazil, 2001)	14	ND	12 (13/108)	ND	
	Cariacica	F1	10^8.7^ MID_50_/mL	74018 (Brazil, 2001)	14	ND	13 (15/119)	ND	
	Boa Vista	F1	10^8.7^ MID_50_/mL	74018 (Brazil, 2001)	14	ND	13 (15/116)	ND	
	Represa do Cigano	F1	10^8.7^ MID_50_/mL	74018 (Brazil, 2001)	14	ND	16 (9/56)	ND	
	Sao Luis	F1	10^8.7^ MID_50_/mL	74018 (Brazil, 2001)	14	ND	20 (22/112)	ND	
	Maringua	F1	10^8.7^ MID_50_/mL	74018 (Brazil, 2001)	14	ND	23 (27/119)	ND	
	Porto Velho	F1	10^8.7^ MID_50_/mL	74018 (Brazil, 2001)	14	ND	24 (29/119)	ND	
	Campo Grande	F1	10^8.7^ MID_50_/mL	74018 (Brazil, 2001)	14	ND	25 (26/104)	ND	
	Potim	F1	10^8.7^ MID_50_/mL	74018 (Brazil, 2001)	14	ND	27 (32/118)	ND	
	Belem	F1	10^8.7^ MID_50_/mL	74018 (Brazil, 2001)	14	ND	34 (37/109)	ND	
	Ananindeua	F1	10^8.7^ MID_50_/mL	74018 (Brazil, 2001)	14	ND	46 (52/112)	ND	
	Foz do Iguaçu	F1	10^8.7^ MID_50_/mL	74018 (Brazil, 2001)	14	ND	49 (53/109)	ND	
	Santos	F2	7–7.8 Log_10_ pfu /mL	71528 MG2001 (Minas Gerais, 2001)	10–14	35 (12/34)	28 (11/39)^e^	ND	[25]
			6.3 Log_10_ pfu /mL	71528 MG2001 (Minas Gerais, 2001)	21	ND	23 (5/22)	20 (4/20)^f^	
Venezuela	Maracay	F1	10^8.7^ MID_50_/mL	74018 (Brazil, 2001)	14	ND	14 (18/132)	ND	[24]
USA	West Palm Beach	F1	10^8.7^ MID_50_/mL	74018 (Brazil, 2001)	14	ND	25 (26/105)	ND	[24]
Cambodia	Phnom Penh	F1	10^8.7^ MID_50_/mL	74018 (Brazil, 2001)	14	ND	64 (67/104)	ND	[24]
Vietnam	Ho Chi Minh city	F1	10^8.7^ MID_50_/mL	74018 (Brazil, 2001)	14	ND	48 (59/123)	ND	[24]
Australia	Cairns	F2	10^7.2^ TCID_50_/mL 10^6.7^ TCID_50_/mL	BA-55 (Nigeria, 1987) OBS 7549 (Bolivia, 1999)	14	80 (20/25) 24 (6/25)	90 (18/20) 100 (6/6)	72 (13/18) 100 (6/6)	[26]
	Townsville	F3	10^7.2^ TCID_50_/mL 10^6.7^ TCID_50_/mL	BA-55 (Nigeria, 1987) OBS 7549 (Bolivia, 1999)	14	72 (18/25) 36 (9/25)	83 (15/18) 89 (8/9)	100 (15/15) 87 (7/8)	
Guadeloupe	Les Abimes	F1	10^6.5^ ffu/mL	4408 (Brazil, 2008)	14	70 (21/30)	57 (12/21)	8 (1/12)	Our study
					21	50 (15/30)	53 (8/15)	12 (1/8)	

^a^ Generation

^b^ Infection rate

^c^ Dissemation rate

^d^ Transmission rate

^e^ Dissemination efficiency

^f^ Transmission efficiency.

The pattern of *Ae*. *aegypti* Guadeloupe infected with a YFV belonging to the 1E lineage (IEC-4408; [10]) should be close to the profile of mosquitoes from the American continent. *Ae*. *aegypti* from Rio and Manaus presented similar IR, DR and TR when compared to *Ae*. *aegypti* Guadeloupe (see Table 1, [10]). It has been demonstrated previously that *Ae*. *aegypti* from the Caribbean were genetically close to mosquitoes from Brazil [27]. However, other factors should be considered to assess the risk of transmission; while YF is still absent from Asia, the vector competence of Asian *Ae*. *aegypti* (see Table 1; Phnom Penh: DR = 64%, Ho Chi Minh city: DR = 48%) was higher than values of *Ae*. *aegypti* from Africa. These factors include: vector densities, trophic preference of vectors for humans, proportion of immunologically naïve humans, and environmental conditions favorable to transmission. Surveillance of travelers coming from YFV-endemic regions of Africa or South America able to initiate a local transmission in the Caribbean should be reinforced. Likewise, vaccination coverage should be reexamined as the live-attenuated 17D is one of the most effective vaccines available against this arbovirus.

## References

[1] Monath TP, Vasconcelos PF. Yellow fever. Journal of clinical virology: the official publication of the Pan American Society for Clinical Virology. 2015;64:160–73. 10.1016/j.jcv.2014.08.030 . 25453327

[2] Vasconcelos PF. Yellow fever in Brazil: thoughts and hypotheses on the emergence in previously free areas. Rev Saude Publica. 2010;44(6):1144–9. . 21109907 10.1590/s0034-89102010005000046

[3] PAHO. Epidemiological Update: Yellow Fever. 20 March 2018. Washington, D.C.: 2018.

[4] Bryan CS, Moss SW, Kahn RJ. Yellow fever in the Americas. Infect Dis Clin North Am. 2004;18(2):275–92, table of contents. Epub 2004/05/18. 10.1016/j.idc.2004.01.007 . 15145381

[5] Goodyear JD. The sugar connection: a new perspective on the history of yellow fever. Bull Hist Med. 1978;52(1):5–21. Epub 1978/01/01. . 352452

[6] Cathey JT, Marr JS. Yellow fever, Asia and the East African slave trade. Trans R Soc Trop Med Hyg. 2014;108(5):252–7. Epub 2014/04/20. 10.1093/trstmh/tru043 . 24743951

[7] PAHO. The feasibility of eradicating Aedes aegypti in the Americas. Rev Panam Salud Publica. 1997;1(1):68–72. Epub 1997/01/01. . 9128110

[8] Vasconcelos PFC, Rosa APAT, Pinheiro fP, Rodrigues SG, S.T. RE, Cruz ACR, et al Aedes aegypti, Dengue and Re-urbanization of Yellow Fever in Brazil and other South American Countries—Past and Present Situation and Future Perspectives. Dengue Bulletin. 1999;23:11.

[9] Barrett AD, Higgs S. Yellow fever: a disease that has yet to be conquered. Annu Rev Entomol. 2007;52:209–29. 10.1146/annurev.ento.52.110405.091454 . 16913829

[10] Couto-Lima D, Madec Y, Bersot MI, Campos SS, Motta MA, Santos FBD, et al Potential risk of re-emergence of urban transmission of Yellow Fever virus in Brazil facilitated by competent Aedes populations. Sci Rep. 2017;7(1):4848 10.1038/s41598-017-05186-3 . 28687779 PMC5501812

[11] Larrieu S, Cassadou S, Rosine J, Chappert JL, Blateau A, Ledrans M, et al Lessons raised by the major 2010 dengue epidemics in the French West Indies. Acta Trop. 2014;131:37–40. Epub 2013/12/10. 10.1016/j.actatropica.2013.11.023 . 24315801

[12] Dorleans F, Hoen B, Najioullah F, Herrmann-Storck C, Schepers KM, Abel S, et al Outbreak of Chikungunya in the French Caribbean Islands of Martinique and Guadeloupe: Findings from a Hospital-Based Surveillance System (2013–2015). Am J Trop Med Hyg. 2018;98(6):1819–25. Epub 2018/04/26. 10.4269/ajtmh.16-0719 . 29692295 PMC6086161

[13] Daudens-Vaysse E, Ledrans M, Gay N, Ardillon V, Cassadou S, Najioullah F, et al Zika emergence in the French Territories of America and description of first confirmed cases of Zika virus infection on Martinique, November 2015 to February 2016. Euro Surveill. 2016;21(28). Epub 2016/07/23. 10.2807/1560-7917.ES.2016.21.28.30285 . 27447300

[14] Gustave J. [The prevention of dengue in Guadeloupe]. Bull Soc Pathol Exot. 1996;89(2):143–4. Epub 1996/01/01. . 8924773

[15] Goindin D, Delannay C, Gelasse A, Ramdini C, Gaude T, Faucon F, et al Levels of insecticide resistance to deltamethrin, malathion, and temephos, and associated mechanisms in Aedes aegypti mosquitoes from the Guadeloupe and Saint Martin islands (French West Indies). Infect Dis Poverty. 2017;6(1):38 Epub 2017/02/12. 10.1186/s40249-017-0254-x ; PubMed Central PMCID: PMCPMC5303256. 28187780 PMC5303256

[16] Gustave J, Fouque F, Cassadou S, Leon L, Anicet G, Ramdini C, et al Increasing Role of Roof Gutters as Aedes aegypti (Diptera: Culicidae) Breeding Sites in Guadeloupe (French West Indies) and Consequences on Dengue Transmission and Vector Control. J Trop Med. 2012;2012:249524 Epub 2012/05/02. 10.1155/2012/249524 ; PubMed Central PMCID: PMCPMC3323855. 22548085 PMC3323855

[17] Dubrulle M, Mousson L, Moutailler S, Vazeille M, Failloux AB. Chikungunya virus and *Aedes* mosquitoes: saliva is infectious as soon as two days after oral infection. PLoS One. 2009;4(6):e5895 10.1371/journal.pone.0005895 ; PubMed Central PMCID: PMC2690823. 19521520 PMC2690823

[18] Payne AF, Binduga-Gajewska I, Kauffman EB, Kramer LD. Quantitation of flaviviruses by fluorescent focus assay. J Virol Methods. 2006;134(1–2):183–9. 10.1016/j.jviromet.2006.01.003 . 16510196

[19] Pastorino BA, Peyrefitte CN, Almeras L, Grandadam M, Rolland D, Tolou HJ, et al Expression and biochemical characterization of nsP2 cysteine protease of Chikungunya virus. Virus Res. 2008;131(2):293–8. 10.1016/j.virusres.2007.09.009 . 17961784 PMC7114110

[20] Lambrechts L, Scott TW, Gubler DJ. Consequences of the expanding global distribution of Aedes albopictus for dengue virus transmission. PLoS Negl Trop Dis. 2010;4(5):e646 10.1371/journal.pntd.0000646 ; PubMed Central PMCID: PMC2876112. 20520794 PMC2876112

[21] Ellis BR, Sang RC, Horne KM, Higgs S, Wesson DM. Yellow fever virus susceptibility of two mosquito vectors from Kenya, East Africa. Trans R Soc Trop Med Hyg. 2012;106(6):387–9. Epub 2012/04/24. 10.1016/j.trstmh.2012.02.007 . 22521217

[22] Jupp PG, Kemp A. Laboratory vector competence experiments with yellow fever virus and five South African mosquito species including Aedes aegypti. Transactions of the Royal Society of Tropical Medicine and Hygiene. 2002;96(5):493–8. . 12474475 10.1016/s0035-9203(02)90417-7

[23] Vazeille M, Yebakima A, Lourenco-de-Oliveira R, Andriamahefazafy B, Correira A, Rodrigues JM, et al Oral receptivity of *Aedes aegypti* from Cape Verde for yellow fever, dengue, and chikungunya viruses. Vector Borne Zoonotic Dis. 2013;13(1):37–40. 10.1089/vbz.2012.0982 . 23199267

[24] Lourenco-de-Oliveira R, Vazeille M, Bispo de Filippis AM, Failloux AB. Oral susceptibility to yellow fever virus of *Aedes aegypti* from Brazil. Mem Inst Oswaldo Cruz. 2002;97(3):437–9. . 12048581 10.1590/s0074-02762002000300031

[25] Johnson BW, Chambers TV, Crabtree MB, Filippis AM, Vilarinhos PT, Resende MC, et al Vector competence of Brazilian Aedes aegypti and Ae. albopictus for a Brazilian yellow fever virus isolate. Transactions of the Royal Society of Tropical Medicine and Hygiene. 2002;96(6):611–3. . 12625133 10.1016/s0035-9203(02)90326-3

[26] van den Hurk AF, McElroy K, Pyke AT, McGee CE, Hall-Mendelin S, Day A, et al Vector competence of Australian mosquitoes for yellow fever virus. The American journal of tropical medicine and hygiene. 2011;85(3):446–51. 10.4269/ajtmh.2011.11-0061 ; PubMed Central PMCID: PMC3163864. 21896802 PMC3163864

[27] Mousson L, Dauga C, Garrigues T, Schaffner F, Vazeille M, Failloux AB. Phylogeography of *Aedes* (Stegomyia) *aegypti* (L.) and *Aedes* (Stegomyia) *albopictus* (Skuse) (Diptera: Culicidae) based on mitochondrial DNA variations. Genetical research. 2005;86(1):1–11. 10.1017/S0016672305007627 . 16181519

